# Structural and Functional Hippocampal Correlations in Environmental Enrichment During the Adolescent to Adulthood Transition in Mice

**DOI:** 10.3389/fnsys.2021.807297

**Published:** 2022-02-15

**Authors:** Francis A. M. Manno, Rachit Kumar, Ziqi An, Muhammad Shehzad Khan, Junfeng Su, Jiaming Liu, Ed X. Wu, Jufang He, Yanqiu Feng, Condon Lau

**Affiliations:** ^1^Center for Imaging Science, Department of Biomedical Engineering, Whiting School of Engineering, Johns Hopkins University, Baltimore, MD, United States; ^2^Department of Physics, City University of Hong Kong, Hong Kong, Hong Kong SAR, China; ^3^Perelman School of Medicine, University of Pennsylvania, Philadelphia, PA, United States; ^4^Medical Scientist Training Program, University of Pennsylvania, Philadelphia, PA, United States; ^5^Guangdong Provincial Key Laboratory of Medical Image Processing, School of Biomedical Engineering, Southern Medical University, Guangzhou, China; ^6^Guangdong-Hong Kong-Macao Greater Bay Area Center for Brain Science and Brain-Inspired Intelligence, Key Laboratory of Mental Health of the Ministry of Education, Southern Medical University, Guangzhou, China; ^7^F.M. Kirby Neurobiology Center, Boston Children’s Hospital, Harvard Medical School, Harvard University, Boston, MA, United States; ^8^Department of Electrical and Electronic Engineering, The University of Hong Kong, Hong Kong, Hong Kong SAR, China; ^9^Laboratory of Biomedical Imaging and Signal Processing, The University of Hong Kong, Hong Kong, Hong Kong SAR, China; ^10^Department of Neuroscience, City University of Hong Kong, Hong Kong, Hong Kong SAR, China; ^11^Department of Biomedical Sciences, City University of Hong Kong, Hong Kong, Hong Kong SAR, China

**Keywords:** environmental enrichment, rsfMRI, DTI, volumetry, hippocampus

## Abstract

Environmental enrichment is known to induce neuronal changes; however, the underlying structural and functional factors involved are not fully known and remain an active area of study. To investigate these factors, we assessed enriched environment (EE) and standard environment (SE) control mice over 30 days using structural and functional MRI methods. Naïve adult male mice (*n* = 30, ≈20 g, C57BL/B6J, postnatal day 60 initial scan) were divided into SE and EE groups and scanned before and after 30 days. Structural analyses included volumetry based on manual segmentation as well as diffusion tensor imaging (DTI). Functional analyses included seed-based analysis (SBA), independent component analysis (ICA), the amplitude of low-frequency fluctuation (ALFF), and fractional ALFF (fALFF). Structural results indicated that environmental enrichment led to an increase in the volumes of cornu ammonis 1 (CA1) and dentate gyrus. Structural results indicated changes in radial diffusivity and mean diffusivity in the visual cortex and secondary somatosensory cortex after EE. Furthermore, SBA and ICA indicated an increase in resting-state functional MRI (rsfMRI) functional connectivity in the hippocampus. Using parallel structural and functional analyses, we have demonstrated coexistent structural and functional changes in the hippocampal subdivision CA1. Future research should map alterations temporally during environmental enrichment to investigate the initiation of these structural and functional changes.

## Introduction

An enriched environment (EE) induces hippocampal neurogenesis (Kempermann et al., 1997; Clemenson et al., 2015), which results in memory alterations (Greenough et al., 1972), and impedes the effects of senescence due to aging (Greenough et al., 1986). The effects of a simple EE (Hebb, 1947; Sztainberg and Chen, 2010) can lead to upregulation in methyltransferase, known to result in neuronal cell differentiation induced by the nerve growth factor (Rampon et al., 2000). Interestingly, environmental enrichment has been known to delay neurological disorders such as Alzheimer’s disease (AD) and Parkinson’s disease (Nithianantharajah and Hannan, 2006). Despite such research, the causative factors involved in an EE eliciting these neurobiological changes are not currently known. Furthermore, the neuronal processes surrounding enrichment are not fully understood. Mouse resting-state functional MRI (rsfMRI) methods offer a reliable and accurate manner to establish functional connectivity changes (Jonckers et al., 2011; Grandjean et al., 2020). Recently, it was demonstrated that AD transgenic mice raised in an EE show increased connectivity between cornu ammonis (CA1) and cortical areas compared with control mice raised in the standard environment (SE) (Little et al., 2012). An environmental enrichment eliciting changes in AD mice reinforces the idea that enrichment may lead to resilience in the face of disease, which would otherwise severely affect structural and rsfMRI connectivity (Jonckers et al., 2015; Asaad and Lee, 2018).

Previously, structural methods have been used to determine volumetric changes (Li et al., 2020), and diffusion tensor imaging (DTI) has been used to assess microstructural fractional anisotropic changes related to white matter fiber tracts (Scholz et al., 2015). Recently, functional MRI paradigms have been used to assess sensory systems (e.g., somatosensory, olfactory, and auditory; Chen et al., 2020), awake Go/No-Go tasks (Han et al., 2019), and awake behaving fMRI studies (Fonseca et al., 2020; Tsurugizawa et al., 2020). Categorization of structural and functional changes in mice in the above studies, and under differing conditions, greatly assists advances in neuroimaging and could help reveal the underlying mechanisms of environmental enrichment. In this study, we sought to provide a brief investigation into the structural and functional hippocampal changes due to environmental enrichment over 30 days. We wanted to know what structural changes underlie functional changes due to environmental enrichment. To address these questions, we used volumetry, DTI, and rsfMRI with different processing methods to determine if EE mice were different than age-matched SE control mice.

Based on prior literature, we hypothesized that enrichment would elicit structural and functional changes resulting in strengthening neuronal tracts and/or region of interest (ROI) enlargement/reduction, which could be measured using DTI, and volumetry, as well as functional changes, were measured using rsfMRI connectivity metrics. For analysis, *t*-tests were used for DTI and volumetry changes. For seed-based analysis (SBA), independent components analysis (ICA), the amplitude of low-frequency fluctuation (ALFF), or fractional amplitude of low-frequency fluctuation (f/ALFF) analyses, *F*-tests, and *t*-test were applied, and correlation coefficients were calculated for EE vs. SE controls for the hippocampus (primary ROI) and caudate putamen (control region). Each test for each metric was performed to test the null hypotheses that EE mice post-intervention were not significantly different from SE controls or themselves before enrichment against the alternative hypothesis that there was a difference between the compared groups. Our results indicate an enlarged hippocampal volume of CA1 and dentate gyrus (DG) as well as an increase in resting-state local connectivity after EE when compared with SE controls. The structural and functional changes were primarily located within the CA1 hippocampal subdivision. Future studies should explore the underlying environmental factors eliciting the neuronal changes in an EE (colors of objects and number of toys).

## Materials and Methods

**FIGURE 1:**
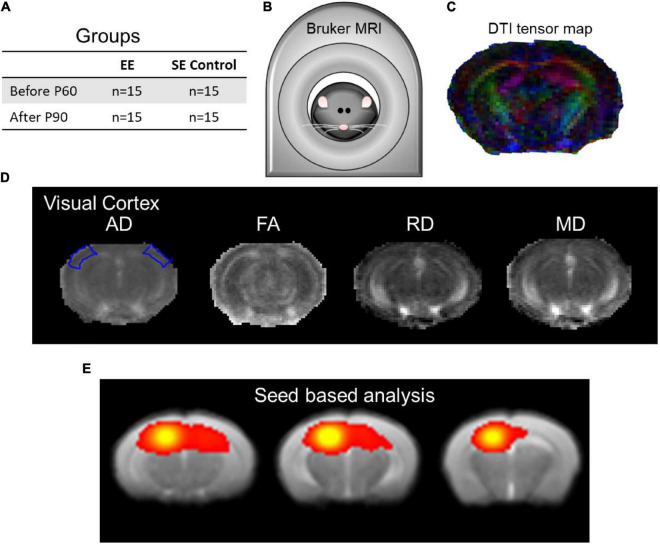
The experimental design setup and data analysis. **(A)** Enriched environment (EE) and standard environment (SE) control groups were assessed using structural and functional MRI. **(B)** Bruker BioSpec 7T scanner with the putative mouse inside. **(C)** Demonstrative DTI tensor color map. **(D)** DTI indices maps (AD, FA, RD, and MD) of the visual cortex at approximately Bregma AP-2.8/–2.9 (Franklin and Paxinos, 2008, Figure 54/55). **(E)** Seed-based analysis (SBA) of local connectivity outlining the hippocampal complex as part of rsfMRI (approximately Bregma AP-3.25/–2.50/–1.75 corresponding to Franklin and Paxinos, 2008, Figures 58/51/45).

Figure 1 and Supplementary Figure 1 illustrate a detailed explanation of the experimental design.

### Animals

Experiments were approved by the City University of Hong Kong Committee on the Use of Live Animal in Teaching and Research in accordance with *The Guide* (National Institutes of Health Guide for the Care and Use of Laboratory Animals, revised 2011). Adult male mice [*n* = 30, ≈20 g, C57BL/B6J, postnatal day 60 (P60)] were used for two MRI repeated sessions over 30 days. The P60-aged mice were chosen for our MRI scanning experiment as they approximately represent the periods of juvenile and entrance into young adulthood (P60 for the initial before scan and P90 for the after scan; Dutta and Sengupta, 2016). All mice were raised and bred from the animal colony at the City University of Hong Kong where they existed in group-caged housing prior to the beginning of the experiment.

### Environmental Enrichment Setup

A total of 30 naive mice raised in the same animal colony were used for the experiment design. Mice were divided into 2 groups of 15 (Figure 1A): (1) EE and (2) SE mice as the control group, with the size of approximately 1.0 m^2^ for the cages of both groups. Both groups were scanned two times in a pre/post design: before/after enrichment (before EE or after EE) or before/after SE design (before control or after control), separated by 30 days.

Environmental enrichment consisted of the following features: (1) running wheel, (2) toys consisting of blocks and balls, and (3) a plastic maze consisting of 1.5-in. tubing cut and connected into different arrangements (see Supplementary Figure 1). Animals were left in these conditions for 30 days. Cages were cleaned once weekly, and animals were handled only during this time. The mice were otherwise left completely undisturbed. Mice were cage-housed under a constant 25°C temperature and 60–70% humidity under a 12/12-h light/dark cycle; the light phase started at 8 a.m. and the dark phase started at 8 p.m. The room housing the cages and mice was temperature-controlled with mice having *ad libitum* access to food and water. No other mice were included in the housing environment.

### MRI Animal Preparation

The MRI data were acquired using a Bruker BioSpec 7T scanner (70/16 PharmaScan, Bruker Biospin, Ettlingen, Germany) with a maximum gradient of 360 mT/m using a transmit-only birdcage coil in combination with an actively decoupled receive-only surface coil. The animals were initially anesthetized in a box with 0–0.3% isoflurane, a bolus of muscle relaxant (pancuronium bromide, 0.1 mg/kg), and subsequently a subcutaneous injection of dexmedetomidine 0.04 mg/kg/h. Throughout MRI scanning, animals were mechanically ventilated at a rate of 80 min^–1^ with 1–3.5% isoflurane in room-temperature air using a ventilator (TOPO, Kent Scientific Corp., Torrington, United States). During echo-planar functional imaging, isoflurane was adjusted to 0.2% upon initiation of the sequence. Animals were placed on a plastic cradle, fixing the head using a tooth bar and plastic screws in the ear canals (Figure 1B). Body temperature was maintained using a water circulation system with a rectal temperature ∼37.0°C used as the controlling factor. Continuous physiological monitoring was performed using an MRI-compatible system (SA Instruments, Stony Brook, NY, United States). Vital signs were within normal physiological ranges (rectal temperature, 36.5–37.5°C; heart rate, 350–420 beats/min; breathing, > 80–100 breaths/min; oxygen saturation, ∼95% with a pulse oximeter) throughout the experiment (Chan et al., 2009; Lau et al., 2011; Cheung et al., 2012a,b; Zhou et al., 2014; Manno et al., 2019, 2021). After the mouse was placed in the scanner, scout images were acquired along the axial, coronal, and sagittal views to position MRI slices accurately. The scanning geometry was positioned according to the Allen Mouse Brain Atlas (Lau et al., 2008)^1^ and the Franklin and Paxinos (2008) atlas. Anatomical images were subsequently acquired using a rapid acquisition refocusing echo T2 (TurboRARE) scan. The sequence order for image acquisition was a T2 for manual volumetry, a DTI scan for structural analysis, and then a series of gradient-echo echo-planar images for resting state.

### Hippocampal Volumetry

The hippocampal complex was localized using MRI slices from the Franklin and Paxinos stereotaxic atlas (2008) at approximately Bregma AP-2.6/–2.7 (first row; Franklin and Paxinos, 2008, Figure 52/53 Bregma –2.54/–2.70 mm), AP-2.8/–2.9 (second row; Franklin and Paxinos, 2008, Figure 54/55 Bregma –2.80/–2.92 mm), and AP-3.0/3.1 (third row; Franklin and Paxinos, 2008, Figure 55/56 Bregma –2.92/–3.08 mm). Hippocampi were delineated into four subregions (CA1, CA2, CA3, and DG) for volumetric analysis. The MRI slices from the aforementioned Bregma AP marks were delineated manually by drawing the hippocampus subdivisions using the T2 image map. The ventricular volume was measured using the T2 image. Volume was calculated by using an estimate from volume (mm^3^) = each area (mm^2^) × (slice thickness + gap among images, in mm). The localization and delineation used the mouse brain atlas ROI as the reference standard (Franklin and Paxinos, 2008). The volume of each hippocampal subdivision was determined and compared with the time points before and after in the EE and the SE control group.

### Diffusion Tensor Imaging Structural Analysis

T2-weighted images (T2WI) in both groups were normalized using SPM12 (The Wellcome Centre for Human Neuroimaging, UCL Queen Square Institute of Neurology, London, United Kingdom). The DTI protocol was followed from our previous study (Manno et al., 2019). The DTI scan sequence was a spin-echo 4-shot echo-planar imaging sequence with 15 diffusion gradient directions, *b*-value is 1,000 s/mm^2^, and five images without diffusion sensitization (*b* = 0.0 ms/μm^2^, b0 images). Images with motion artifacts were discarded. The imaging parameters were repetition time/echo time = 2,000/24.5 ms, δ/Δ = 4/12 ms, field of view = 2.56 × 2.56 cm^2^, data matrix = 96 × 96, slices = 20, slice thickness 0.75 mm, spatial resolution 0.26 × 0.26 mm^2^, and number of excitements = 4. The diffusion-weighted images and b0 images were processed using SPM12 (The Wellcome Centre for Human Neuroimaging, UCL Queen Square Institute of Neurology, London, United Kingdom) and custom Matlab (The Mathworks, Natick, MA, United States) scripts. The mouse brain was manually masked based on the diffusion-weighted image (Figure 1D). Diffusion-weighted images were first registered to the respective b0 image using AIR version 5.25 (Roger Woods, UCLA, United States), and images with severe ghosting were excluded. Distortions in the DTI images were spatially corrected by non-linear registration to the T2-structural image of the same mouse. The parameters of the three transformations were merged into a single transformation (Li et al., 2010). The DTI index maps were calculated by fitting the diffusion tensor model to the diffusion data at each voxel using DTIStudio version 3.02 (The Johns Hopkins University, Baltimore, MD, United States) as previously detailed (Manno et al., 2019). The normalized maps were smoothed with a 0.3 mm Gaussian kernel. Mean diffusivity (MD), fractional anisotropy (FA), axial diffusivity (AD), and radial diffusivity (RD) maps were calculated. Using SPM12 (The Wellcome Centre for Human Neuroimaging, UCL Queen Square Institute of Neurology, London, United Kingdom), the T2-weighted images from individual mice were coregistered to a customized reference brain template with a 3D rigid-body transformation, and the resulting transforming matrix was then applied to register the respective DTI index maps. Regionally, changes of AD, FA, RD, and MD in the hippocampus (HP: Franklin and Paxinos, 2008, Figure 48 Bregma –2.06 mm), visual cortex (VC: Franklin and Paxinos, 2008, Figure 55 Bregma –2.92 mm), auditory cortex (AC: Franklin and Paxinos, 2008, Figure 55 Bregma –2.92 mm), the primary somatosensory cortex (S1: Franklin and Paxinos, 2008, Figure 34 Bregma –0.34 mm), the secondary somatosensory cortex (S2: Franklin and Paxinos, 2008, Figure 31 Bregma 0.02 mm), and the caudate putamen (CPu: Franklin and Paxinos, 2008, Figure 27 Bregma 0.50 mm) were all measured in the EE and SE controls for both time points assessed (before and after).

### Resting-State Functional MRI Acquisition and Processing

A T2 TurboRARE anatomical image [TR, 2,500 ms; TE, 35 ms; matrix size, 256 × 256; field of view (FOV), 16 mm × 16 mm; slice thickness, 500 μm; slices, 22; average, 2; resolution, 62.5 μm × 62.5 μm] was acquired for coregistration. After a local shimming was applied, single-shot echo-planar imaging (EPI) images were acquired to assess rsfMRI. The rsfMRI images were acquired using a gradient-echo echo-planar image (GE-EPI) sequence with the following parameters: FOV = 25.6 mm × 25.6 mm, in-plane resolution 0.4 mm × 0.4 mm with a slice thickness of 0.75 mm, data matrix = 64 × 64, TR = 1,000 ms, TE = 19 ms, 600 acquisitions, two dummy scans. Coregistration of anatomical images was performed using the Allen Brain Institute reference atlas (Lau et al., 2008; see text footnote 1) and the Franklin and Paxinos (2008) atlas. The EPI scan geometry was imported from the anatomical scan geometry. For each rsfMRI session, all images were first corrected for slice timing differences and then realigned to the first image of the series using the SPM12 affine transformation (The Wellcome Centre for Human Neuroimaging, UCL Queen Square Institute of Neurology, London, United Kingdom). A voxel-wise linear detrending with least-squares estimation was performed temporally to eliminate the baseline drift caused by physiological noise and system instability. Spatial smoothing was performed with a 0.5 mm FWHM Gaussian kernel. Motion correction denoising consisted of 12 motion parameters (roll, pitch, yaw, translation in three dimensions, and the first derivative of each of those six parameters). Temporal band-pass filtering was applied with cutoff frequencies at 0.005 and 0.1 Hz. The first 15 image volumes and the last 15 image volumes of each session were discarded to eliminate possible non-equilibrium effects. Finally, high-resolution anatomical images from individual animals were coregistered to a brain template with a 3D rigid-body transformation; the transforming matrix was then applied to the respective rsfMRI data (Manno et al., 2019). SBA, independent component analysis (ICA), ALFF, and fALFF were performed to determine the functional connectivity changes before and after environmental enrichment.

### Resting-State Functional MRI Connectivity Analysis

Two 3 × 3 voxel regions were chosen as the ipsilateral and contralateral seeds, respectively, in the hippocampus and the CPu. Regionally averaged time course from the voxels within each seed served as the respective reference time course. Pearson correlation coefficients were calculated between the reference time course and the time course of every other voxel to generate two rsfMRI connectivity maps for each region. A 3 × 3 voxel region on the contralateral side of the seed was defined as the ROI. Interhemispheric functional connectivity for each region was then quantified by averaging the correlation coefficient value of the corresponding ipsilateral and contralateral ROIs. For ICA, rsfMRI data were analyzed using the GIFT version 2.0h toolbox. The estimated number of components for all rsfMRI data was found to be 20 by the minimum description length (MDL) criterion. The Infomax algorithm was used, and group-level ICA was performed on all rsfMRI data from the same group and at the same time point. The group-level spatial ICA maps of independent resting-state networks were then scaled to *z*-scores and visually inspected to identify the brain regions. The hippocampal network and CPu network were identified based on the spatial patterns about known anatomical and functional locations. For ALFF analysis, the Data Processing Assistant for Resting-State fMRI (DPARSF) toolbox was used (Chao-Gan and Yu-Feng, 2010). The time series for each voxel was transformed to the frequency domain, and the power spectrum was then obtained. Since the power of a given frequency is proportional to the square of the amplitude of this frequency component, the square root was calculated at each frequency of the power spectrum and the averaged square root was obtained across 0.01–0.08 Hz at each voxel. This averaged square root was taken as the ALFF. For fALFF, a ratio of the power of each frequency at the low-frequency range to that of the entire frequency range was considered.

## Results

The structural and functional MRI results reveal that volumetric enlargement and functional connectivity increases occurred in the hippocampus after EE compared with age-matched SE controls and compared with the previous scanning time point 30 days before.

### Structural Changes Resulting From Environmental Enrichment

**FIGURE 2:**
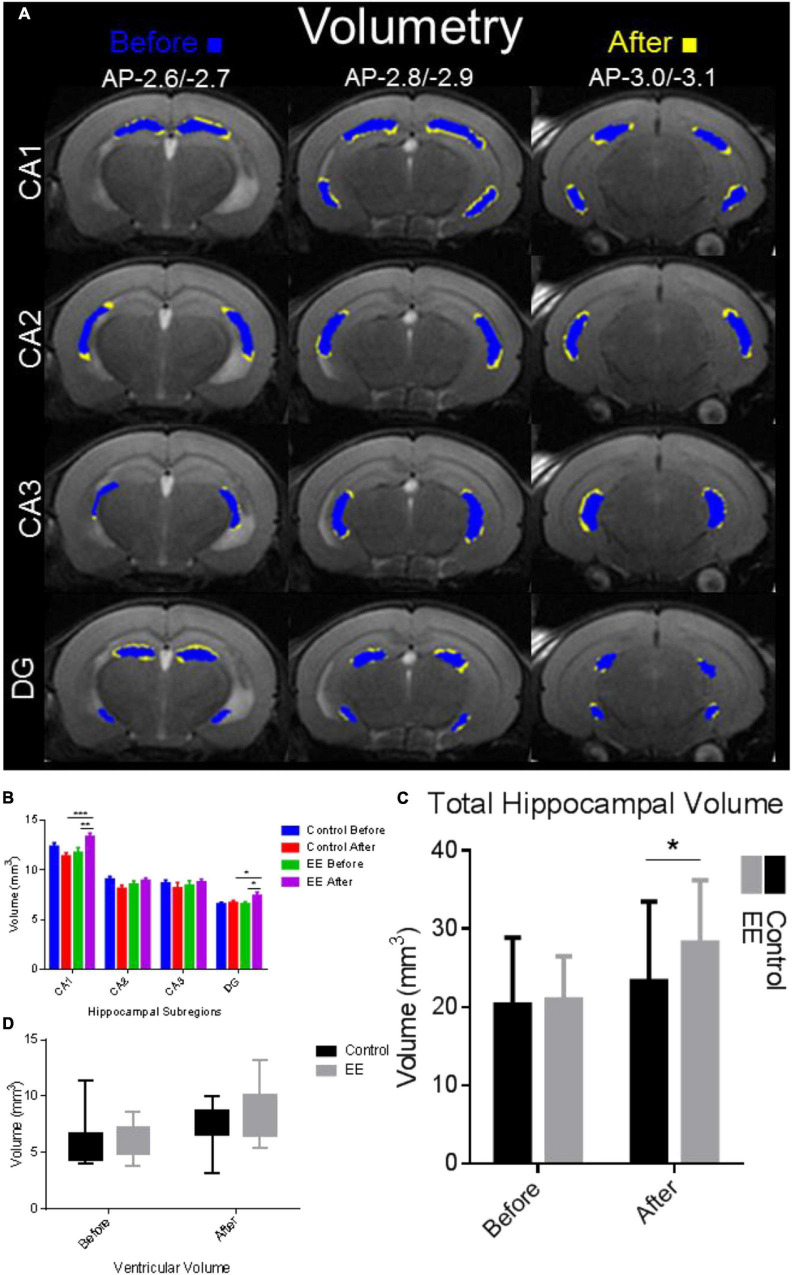
Hippocampal complex volume alterations due to environmental enrichment. **(A)** Volumetry for hippocampal subdivisions. Blue indicates the time point before and yellow indicates the time point after 30 days of EE and SE. The hippocampal complex was delineated manually using the T2 image map from slices AP-2.6/–2.7, AP-2.8/–2.9 to AP-3.0/3.1 (first, second, third rows, respectively; Franklin and Paxinos, 2008). The volume (in mm^3^) of each hippocampal subdivision was determined and compared with the time points before and after in the EE group and SE control group. **(B)** Volume delineations from the hippocampal subdivisions in mm^3^. CA1 volume was significantly increased for the after-EE time point compared with the before EE time point (***p < 0.001), in addition to the control before/after time points (**p < 0.01). DG volume was significantly increased for the after-EE time point compared with the before-EE time point (*p < 0.05), in addition to the control before/after time points (*p < 0.05). **(C)** Total hippocampal volume was significantly increased in the after-EE group compared with the SE control group (*p < 0.05). **(D)** Ventricular volume was enlarged in the after-EE group, although insignificantly compared with the after-SE control group.

Hippocampal volume was significantly enlarged in the after EE time point compared with SE controls and before-EE time point (Figure 2A). Hippocampal volume subdivisions CA1 and DG increased significantly in the after-EE group compared with the after-SE control group, as well as the before-EE and before-SE time point (paired *t*-test *p* < 0.05). The cumulative hippocampal volume found after EE was significantly different than the cumulative volume found for the after-SE time point (paired *t*-test: *t* = 2.205, *df* = 12.6, *p* = 0.0438; Figure 2C). Interestingly, ventricular volume was insignificantly enlarged in the after-EE time point compared with the after-SE time point (paired *t*-test: *t* = 1.858, *df* = 12.7, *p* = 0.0788; Figure 2D). Notably, the ventricular volumes from both groups were within previously reported volumes (4.80 ± 0.40 mm^3^ for 9-week-old C57BL/6J mice Badea et al., 2007). Overall, the hippocampus of mice after EE was enlarged with changes to CA1 accounting for much of the volume increase. We note that the hippocampi from both groups were within previously reported volumes (21 ± 0.5 mm^3^ for 8-week-old male inbred 129S1/SvImJ mice, Kovacević et al., 2005; 25.7 ± 1.1 mm^3^ for 12–14-week-old C57BL/6J male mice, Ma et al., 2005; 25.75 ± 1.04 mm^3^ for 9-week-old C57BL/6J mice Badea et al., 2007; several strains, Wahlsten et al., 2003).

### Diffusion Tensor Imaging Changes Resulting From Environmental Enrichment

**FIGURE 3:**
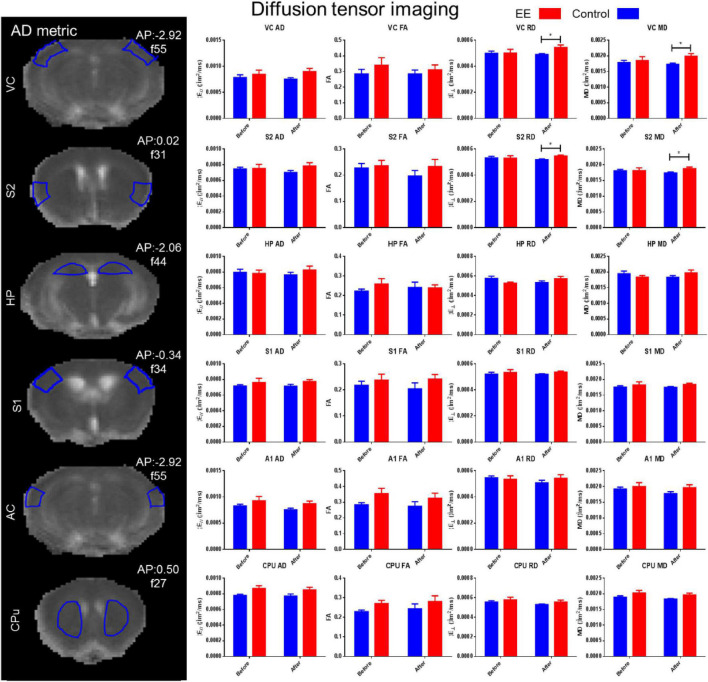
DTI alterations in environmental enrichment. DTI was measured by AD, FA, RD, and MD. There was a significant increase in RD and MD in the visual cortex and secondary somatosensory cortex in the after-EE time point (*t*-test, *p* < 0.05). DTI metrics were assessed in the hippocampus (HP: Bregma –2.06 mm), visual cortex (VC: Bregma –2.92 mm), auditory cortex (AC: Bregma –2.92 mm), the primary somatosensory cortex (S1: Bregma –0.34 mm), and secondary somatosensory cortex (S2: Bregma 0.02 mm), and the caudate putamen (CPu; Bregma 0.50 mm) were all measured in the EE and SE controls for both time points assessed (before and after). Tractography was attempted; however, considerable noise prevented its inclusion in the analyses (see Supplementary Figure 2).

Changes in MD, FA, AD, and RD in the HP, VC, AC, S1, S2, and CPu were determined in the EE and SE controls for both the before and after time points using DTI (see Figure 3). There was a significant increase in RD (paired *t*-test: *t* = 2.148, *df* = 41, *p* = 0.0377) and MD (paired *t*-test: *t* = 2.319, *df* = 41, *p* = 0.0255) in the VC in the after-EE time point compared with the before-EE time point. There was a significant increase in RD (paired *t*-test: *t* = 2.239, *df* = 24, *p* = 0.0347) and MD (paired *t*-test: *t* = 2.020, *df* = 24, *p* = 0.0547) in the S2 in the after-EE time point compared with the before-EE time point. The DTI analysis did not reveal any further significant changes in the ROI possibly due to a low signal-to-noise ratio (SNR) in the presence of high noise (Supplementary Figure 2).

### Resting-State Functional Changes Resulting From Environmental Enrichment

**FIGURE 4:**
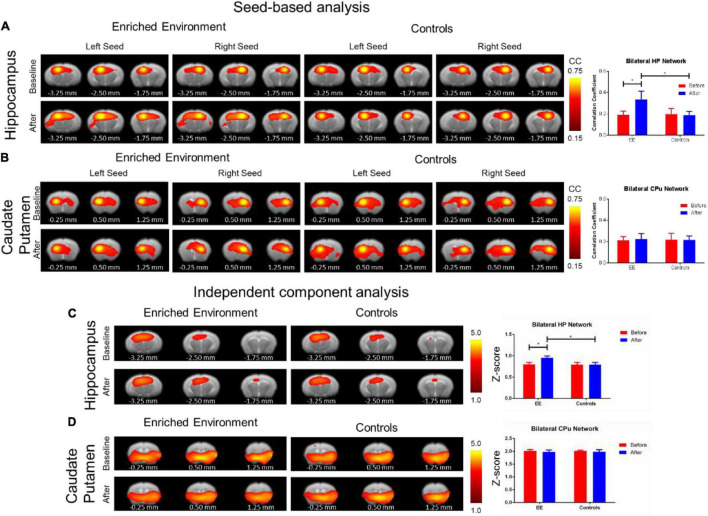
Resting-state functional alterations from environmental enrichment. The SBA analysis: The rsfMRI connectivity change before and after EE compared with the SE controls. **(A)** Functional connectivity maps (left) of the hippocampal (HP) network resulting from SBA for right and left hemisphere seed. Bar plots of average functional connectivity in HP (right) before (red) and after (blue) environmental enrichment. There was a significant increase in hippocampal functional connectivity after environmental enrichment compared with the naïve period as well as compared with the SE controls. **(B)** Functional connectivity maps (left) of the CPu network resulted from SBA for the right and left hemisphere seed. Bar plots of the average functional connectivity in CPu (right) before (red) and after (blue) the EE. In contrast to the hippocampal network, the Cpu network remained relatively unchanged after the EE. Color bar represents correlation coefficient from 0.75 yellow to 0.45 red. The asterisk * indicates significant *p* < 0.05 based on the *F*-test. The ICA analysis: The rsfMRI connectivity change before and after the EE compared with the SE controls. **(C)** Functional connectivity maps (left) of the hippocampal (HP) network resulted from ICA. Bar plots of average *z*-scores in HP (right) before (red) and after (blue) environmental enrichment. There was a significant increase in the hippocampal functional connectivity after environmental enrichment compared with the naïve period as well as compared with the SE controls. **(D)** Functional connectivity maps (left) of the CPu network resulted from ICA. Bar plots of average *z*-scores in Cpu (right) before (red) and after (blue) environmental enrichment. In contrast to the hippocampal network, the CPu network remained relatively unchanged after environmental enrichment. The color bar represents *z*-scores from 5.0 yellow to 1.0 red. The asterisk * indicates significant *p* < 0.05 based on the *F*-test. Bregma AP coordinates are listed below each brain slice.

Each of the other connectivity analyses yielded differing results. The SBA rsfMRI connectivity assessment found a significant increase in the hippocampal functional connectivity in the after-EE compared with before-EE (repeated ANOVA F-Test: *F* = 4.459, *df* = 7.9, *p* = 0.0416; paired *t*-test: *t* = 1.842, *df* = 16, *p* = 0.0841) as well as compared with SE controls (repeated ANOVA *F*-test: *F* = 4569, *df* = 7.16, *p* < 0.0001; paired *t*-test: *t* = 2.729, *df* = 23, *p* = 0.0120; Figure 4A). In contrast to the hippocampal network, the CPu network remained relatively unchanged after environmental enrichment (Figure 4B). The ICA rsfMRI connectivity assessment found a significant increase in the hippocampal functional connectivity in the after-EE compared with the before-EE (repeated ANOVA *F*-test: *F* = 1.480, *df* = 21.12, *p* < 0.4888; paired *t*-test: *t* = 2.400, *df* = 33, *p* = 0.0222) as well as compared with the SE controls (repeated ANOVA *F*-test: *F* = 2566, *df* = 21.29, *p* < 0.0001; paired *t*-test: *t* = 4.410, *df* = 50, *p* < 0.0001; Figure 4C); however, no significant change was found in the CPu network (Figure 4D). No significant ALFF changes in the after-EE or the SE controls were found in the hippocampus or CPu (Supplementary Figure 3A). The f/ALFF and ALFF analyses had considerable noise even after robust denoising procedures similar to SBA and ICA. Despite the noise, a significant increase in both hippocampal and CPu f/ALFF in the after-EE compared with the before EE as well as compared with the SE controls was found (Supplementary Figure 3B). However, we chose not to include f/ALFF and ALFF due to the significant noise present, which likely confounded the results and are more difficult to interpret since our control region CPu was also increased (see Supplementary Figure 3). Both SBA and ICA analyses revealed a significant hippocampal connectivity increase in the after-EE compared with the before-EE and SE controls.

## Discussion

Environmental enrichment is known to lead to neurogenesis (van Praag et al., 1999; Kempermann et al., 2015; Overall and Kempermann, 2018). The process of neurogenesis combines multiple genetic influences and tight connections between multiple system levels of gene expression influenced by the environment (Overall and Kempermann, 2018). The environmental factors or features that initiate this cascade are not generally known (van de Weerd, 1996). In this study, we used functional and structural MRI to assess the structural and functional changes in a group of P60 mice both before and after 30 days in their environments such as SE control and EE (enriched). We found structural and functional changes in the hippocampal subregion CA1 after the 30-day enrichment period.

This study concentrated on the hippocampus as it is known to be modified by environmental enrichment (Kempermann et al., 1997; Clemenson et al., 2015). In the rat, low frequency (1 Hz) hippocampal-cortical oscillatory activity drives brain-wide rsfMRI connectivity likely feeding back to affect sensory cortical processing (Chan et al., 2017). In an EE, this afferent feedback may significantly alter the hippocampus, enlarging in size and increasing the strength of functional connectivity between the hippocampus and senses that intimately interact with the environment. Structural changes in myelination occur rapidly within the first 3 months of mouse life, but volume appears to be stable by 3 weeks (Hammelrath et al., 2016). This is interesting to note as our data indicate hippocampal volume was not significantly different between the time points before and after for SE control mice, confirming stable volume during this period.

A C58/J inbred mouse model of restricted, repetitive behavior found reduced volume in the cortical and basal ganglia regions implicated in repetitive behavior, including the motor cortex, striatum, globus pallidus, subthalamic nucleus, and cerebellum (Wilkes et al., 2019). However, these identical areas are implicated in normal volumetric changes due to motor learning (Badea et al., 2019). In this study, we found DTI alterations in S2 due to environmental enrichment. While this may be due to their enriched sensory involvement and not an aberrant factor of being caged (i.e., as in the stereotypy model above), these data support the conclusion that it is not solely increased exercise and movement of mice in an EE that elicits the volumetric changes seen in our EE group. Rather, there may be an environment-brain interaction (i.e., the feedback and the type of environmental features) that contribute, and that environmental enrichment is not purely an exercise effect. It is interesting to note that mouse brain volume of independent areas has the highest mean heritability, whereas FA, MD, AD, and RD are less heritable (Wang et al., 2020). *Ex vivo* mouse DTI (Zhang et al., 2002) has previously shown success in reproducing and depicting neuronal fiber tracts (Johnson et al., 2010; Jiang and Johnson, 2011); however, low SNR in this study could prevent accurate replication of tracts in mouse, possibly due to the repeated-measures design and the nature of *in vivo* imaging. However, DTI and tractography in mice is still a relatively new approach, which should be further pursued (Harsan et al., 2010).

Environmental enrichment significantly affected the rsfMRI functional connectivity in the bilateral hippocampus at large. Prior studies found that the functional connectivity for default mode network, internetwork connections increased in mice up to 8.5 months, and then a significant reduction in the functional connectivity strength was observed due to aging until 12.5 months (Egimendia et al., 2019). In this study, we chose mice aged 60 days and scanned for 30 days as it represented the youth to adulthood transition (Dutta and Sengupta, 2016), where mice experience age-dependent neuronal plasticity (Harburger et al., 2007; Navailles et al., 2008; Hill et al., 2018) and during which the structural and functional metrics are known to be altered in humans (Buimer et al., 2020). The rsfMRI networks are known to be modulated by different physiological states (Chuang and Nasrallah, 2017) such as anesthetic states (Grandjean et al., 2014; Nasrallah et al., 2014; Bukhari et al., 2017, 2018; Wu et al., 2017; Xie et al., 2020), satiety and hunger, i.e., fasting (Tsurugizawa et al., 2019), and stress (Weible et al., 2017; Johnson et al., 2018). In this study, we show that rsfMRI networks are also influenced by an EE over 30 days. The environment in our paradigm included various enrichment options that provided considerable exercise (van Praag et al., 1999; Leise et al., 2013; Grégoire et al., 2014) and nesting opportunities (van de Weerd et al., 1997; Gaskill et al., 2013). Furthermore, rsfMRI networks are a direct result of underlying structural features and likely represent a mouse recapitulation of the human default mode network (Stafford et al., 2014; Liska et al., 2015; Gozzi and Schwarz, 2016; Mills et al., 2018; Melozzi et al., 2019). Such a modification of these functional connectivity networks (as in the previously mentioned studies and as we have seen in our paradigm) indicates the importance of future work in defining attributes of the environment which alter and how these features and factors modify their neuronal counterparts.

There are several limitations in this assessment of mouse structure and functional changes due to environmental enrichment. First, in this preliminary report, we restricted our structural and functional analysis to the hippocampus, and future reports could examine whole-brain-wide structural and functional connectome changes due to environmental enrichment. The sample size in this study was low (*n* = 30, separated by 30 days), which may have contributed to the low SNR of the DTI data or the rsfMRI data quality. Furthermore, we limited the analysis to male mice only to eliminate the effect sizes due to sex (Spring et al., 2007). Future studies should consider increasing the sample size to address data quality concerns as well as imaging multiple time points to develop a better timeline of the alterations observed in this study. Furthermore, our DTI processing pipeline may not have been sensitive enough to detect all of the alterations seen in environmental enrichment (EE) during this short-term period, which could contribute to the failure of tractography to yield consistent results for some regions. Optimizations and improvements in this pipeline and the scanning protocol (e.g., increasing the number of diffusion gradient directions) for subsequent tractography might also allow fiber tract microstructure to be assessed more adequately. It is important to note that the sharing of MRI equipment through “core-facilities” (Hockberger et al., 2018) may inherently limit key considerations for any scanning protocol, some of which include scanning-time, trial-and-error approaches, experimental design, and pulse sequences and, most importantly, scientific tinkering. Future protocol “tinkering” should include these many considerations when attempting interventions and challenges to environmental enrichment in an attempt to understand the mechanisms that lead to alterations in neuronal structure and function metrics due to changing environments.

## Data Availability Statement

The datasets presented in this study can be found in online repositories. The names of the repository/repositories and accession number(s) can be found below: https://francismanno.github.io/fmanno/.

## Ethics Statement

The animal study was reviewed and approved by the Committee on the Use of Live Animal in Teaching and Research of the City University of Hong Kong.

## Author Contributions

Author contributions are indicative of the environmental enrichment project organized by CL, YF, and FM at the City University of Hong Kong. FM, RK, ZA, MK, JS, JL, EW, JH, YF, and CL designed the research and wrote and edited the manuscript. FM and MK performed the research and data curation. FM, MK, and RK analyzed the data. YF and CL funded the research. All authors contributed to the article and approved the submitted version.

## Conflict of Interest

The authors declare that the research was conducted in the absence of any commercial or financial relationships that could be construed as a potential conflict of interest.

## Publisher’s Note

All claims expressed in this article are solely those of the authors and do not necessarily represent those of their affiliated organizations, or those of the publisher, the editors and the reviewers. Any product that may be evaluated in this article, or claim that may be made by its manufacturer, is not guaranteed or endorsed by the publisher.

## Footnotes

1
http://www.brain-map.org
