# Supplementary material for: Structural and Functional Hippocampal Correlations in Environmental Enrichment During the Adolescent to Adulthood Transition in Mice
Source: Front Syst Neurosci. 2022 Feb 15;15:807297. doi: 10.3389/fnsys.2021.807297 (PMC8886042; doi:10.3389/fnsys.2021.807297)
Supplement: Supplementary file 1 [file Data_Sheet_1.docx]

1. **Supplemental information**

**SI-Figure-1 Methods**


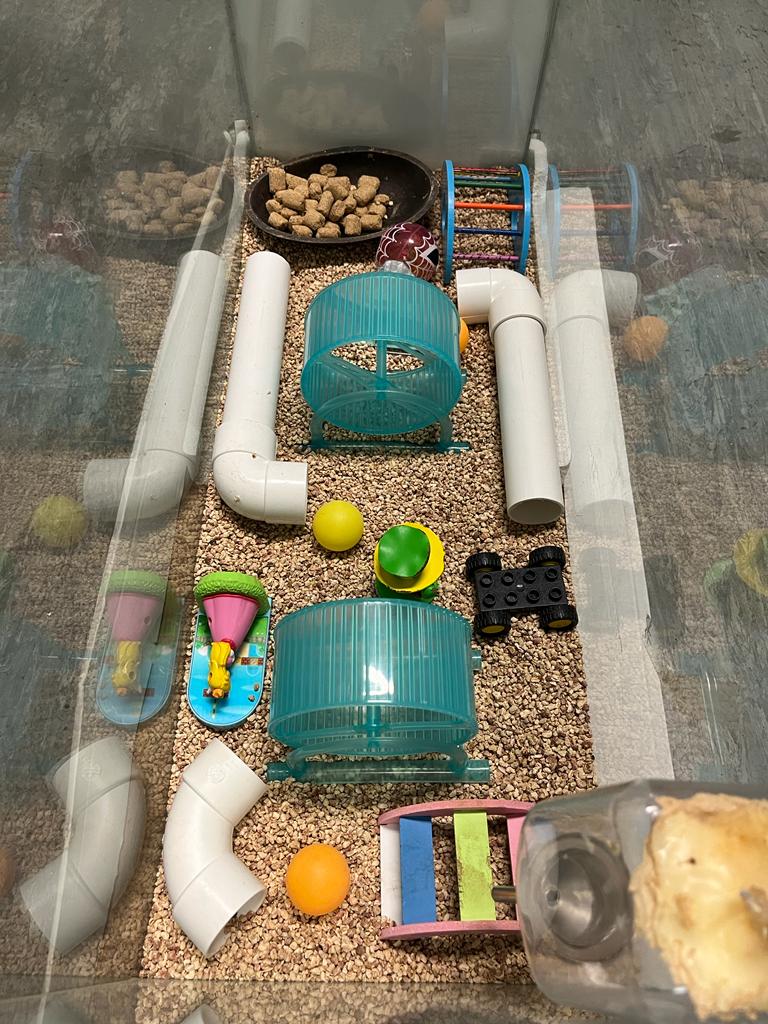

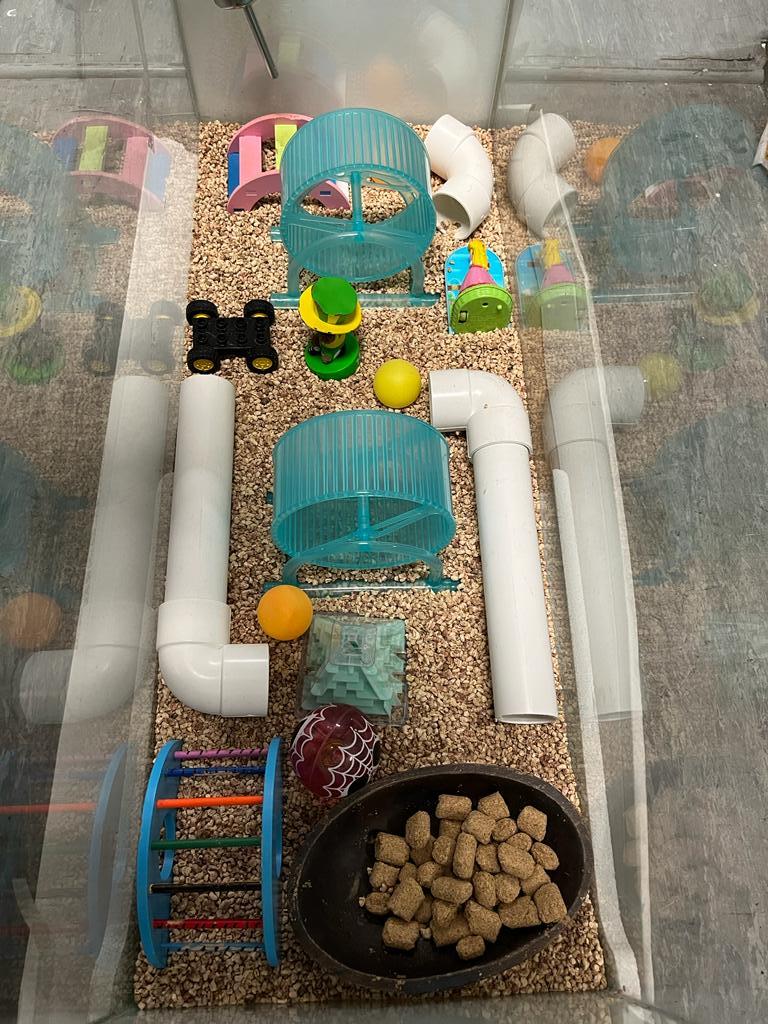


**SI-Figure-1 Environmental enrichment**. Photographs of two environmental enrichment (EE) cage setups. Objects included wheels, tunnels, and various toys.

**SI-Figure-2 DTI tractography**


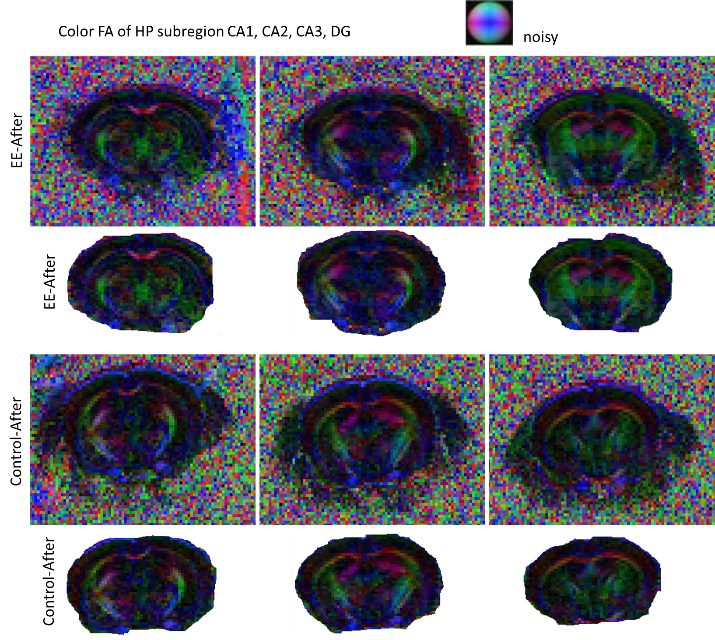


**SI-Figure-2 DTI tractography**. DTI was attempted to determine changing tracts in EE compared to SE control over the 30-day period. DTI eigenvector orientation: blue is superior-inferior, red is left-right, and green is anterior-posterior. Our group attempted DTI tractography, however considerable noise was apparent. Higher SNR or spatial resolution of data was needed for tractography than was originally planned. The small size of the mouse brain contributed to the difficulty for tractography.

**SI-Figure-3 ALFF and f/ALFF rsfMRI**


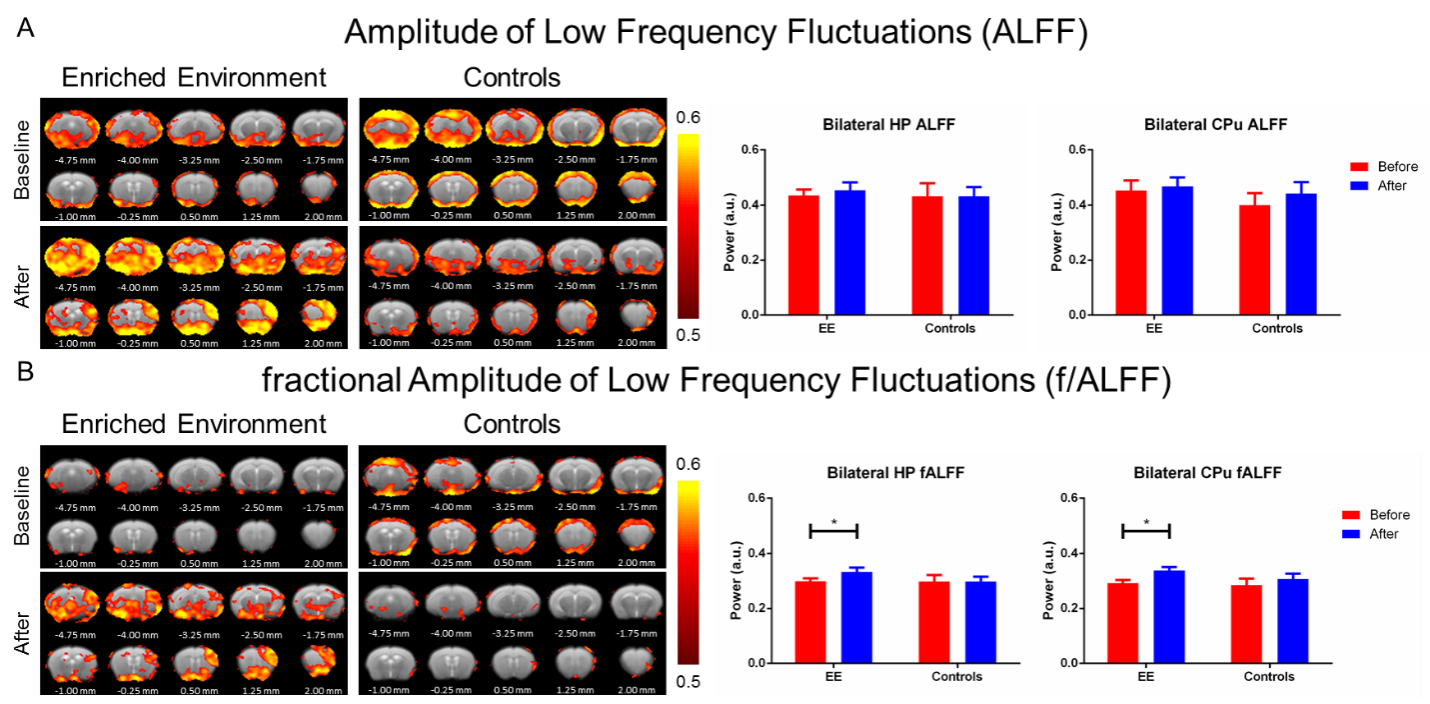


**SI-Figure-3 ALFF and f/ALFF rsfMRI**. A) No significant ALFF changes were found before or after the EE (i.e. mice were identical at both time-points). ALFF maps (left) and bar plots (right) of average power in HP and CPu before (red) and after (blue) environmental enrichment. There were no significant changes in hippocampal and CPu ALFF after environmental enrichment compared to before as well as the controls at both time-points. Color bar represents power (a.u.) from 0.6 yellow to 0.5 red. Two-way ANOVA followed by Bonferroni’s post hoc tests were applied. B) The fALFF analysis found a significant change due to environmental enrichment in HP; however, this was also found in the CPu. fALFF maps (left) and bar plots (right) of average fractional power in HP and CPu before (red) and after (blue) environmental enrichment. There was a significant increase in both hippocampal and CPu fALFF after environmental enrichment. Color bar represents power (a.u.) from 0.6 yellow to 0.5 red. The asterisk * indicates significant p < 0.05. Both ALFF and f/ALFF had considerably more noise than SBA and ICA analyses. Bregma AP coordinates are listed below each brain slice.
